# How does a β-barrel integral membrane protein insert into the membrane?

**DOI:** 10.1007/s13238-016-0273-6

**Published:** 2016-05-28

**Authors:** Xuejun C. Zhang, Lei Han

**Affiliations:** National Laboratory of Macromolecules, National Center of Protein Science- Beijing, Institute of Biophysics, Chinese Academy of Sciences, Beijing, 100101 China

**Table:** 

STRUCTURAL INSIGHT INTO THE ASSEMBLY MECHANISM OF β-BARREL MEMBRANE PROTEINS	
Integral membrane proteins are categorized into two major groups: transmembrane β-helical bundles and β-barrels. β-Barrel membrane proteins are usually located in the outer membranes of Gram-negative bacteria, as well as mitochondria and chloroplasts of eukaryotic cells. The mechanism of membrane insertion of β-barrel membrane proteins is not well understood compared with that of β-helical membrane proteins. Recently reported crystal structures of the β-barrel assembly machinery (Bam) complex from Escherichia coli shed new light on the mechanism of folding and membrane insertion of β-barrel membrane proteins. Expanding upon existing models, Zhang and his colleagues propose and discuss a general mechanism in which the Bam complex provides a chaperone that is specific for the unfolded nascent peptide, a template for the initiation of barrel building, and a channel for the hydrophilic, extracellular loops of the β-barrel to move across the membrane. —Chih-chen Wang*	

Integral membrane proteins (MPs) are categorized into two major groups: transmembrane (TM) α-helical bundles and β-barrels (Cymer et al., 2014). Both types of structures permit the backbones of their peptides to fulfill their hydrogen-bonding potential in a lipid-bilayer environment. The thermal stability of a typical β-barrel MP is usually much higher than that of a helical MP. Thus, β-barrel MPs are able to withstand near boiling-temperatures during purification (Han et al., 2016), compared with the typical melting temperatures of 50°C or lower for α-helical TM proteins. In fact, unfolding a β-barrel MP in a single-molecule assay typically requires multiple steps, each of which requires a force of 100–300 pN (Thoma et al., 2015). Considering the thickness of the membrane as well as the multiple steps, unfolding one mole of β-barrel MP molecules would require ~10^3^ kJ of energy (equivalent to 400 RT or energy from hydrolyzing 20–30 moles of ATP). Such high thermal stability is considered to be the sole source of the energy that drives the folding of β-barrel MPs (Fleming, 2015).

Nearly all α-helical MPs are located in the plasma membrane or its equivalents (e.g., the inner membrane of Gram-negative bacteria and the endoplasmic reticulum membrane of eukaryotic cells). The mechanism of membrane insertion of α-helical MPs is much better understood (Cymer et al., 2014), at least conceptually, than that of β-barrel MPs. Hydrophobic TM helices, usually one pair at a time, insert co-translationally into the membrane, with assistance from translocon machineries (e.g., the Sec, YidC, and Tat complexes) (Gogala et al., 2014; Kumazaki et al., 2014; Widdick et al., 2006). In this case, the translocon provides a TM hydrophilic slot/channel to overcome the kinetic energy barrier of the hydrophobic lipid bilayer, thereby facilitating the movement of the hydrophilic, exo-membrane loops of the helix-hairpins across the membrane. The driving force of the membrane insertion comes from the favorable hydrophobic interaction between the TM helices and the lipid bilayer. The orientations of the TM helices are guided by the positive-inside rule (von Heijne, 1992), which dictates that the more positively-charged ends of TM helices remain on the intracellular side of the membrane, which carries a negative-inside electrostatic potential. For Gram-negative bacteria, only the inner membrane carries a potential, and the co-translational folding system ensures that most (if not all) α-helical MPs reside in the inner membrane. Therefore, to perform its biological functions, the outer membrane (OM) has to exploit a fundamentally different type of integral MP, namely β-barrel MPs (referred to hereafter as OMPs), as well as a different folding mechanism, for OMPs to avoid being stuck in the inner membrane. In addition to their different membrane location compared with α-helical MPs, OMPs must overcome additional energy barriers during folding (Fleming, 2015). After translocation into the periplasmic space, nascent OMPs must remain unfolded and pass through the aqueous periplasm. Like the exo-membrane loop in a helix-hairpin, the extracellular loops (ECLs) of a β-barrel must overcome the energy barrier of the hydrophobic OM during membrane insertion. It has been shown that a thinned membrane allows faster folding and assembly of OMPs (Burgess et al., 2008; Gessmann et al., 2014). In addition, the β-strands of a nascent OMP need to insert sequentially into the OM to avoid misfolding.

For most known structures of OMPs, the β-barrels usually possess an even number of β-strands (Fairman et al., 2011), with both the amino (N)- and carboxyl (C)-termini residing in the periplasmic space (Rollauer et al., 2015). The number of β-strands of known β-barrel structures varies from 8 to 36. For example, OmpA (Pautsch and Schulz, 1998), FhaC (Clantin et al., 2007), FhuA (Ferguson et al., 1998), LptD (Qiao et al., 2014), and CsgG (Cao et al., 2014) contain eight, 16, 22, 26, and 36 (i.e., 4 × 9) strands, respectively (Note that the voltage-dependent anion channel of the mitochondrial OM is known to have 19 β-strands (PDB ID: 3EMN) (Ujwal et al., 2008).). In addition, extracellular loops are usually longer than periplasmic loops. Furthermore, the outside surface of a β-barrel is hydrophobic, while the hydrophobicity and charge distribution inside the cavities of β-barrels vary from protein to protein. Thus, each strand of a β-barrel often shows a pattern of alternating hydrophobic and hydrophilic residues. The hydrophobic residues will eventually form the surface of the β-barrel that faces the lipid bilayer.

**Figure 1:**
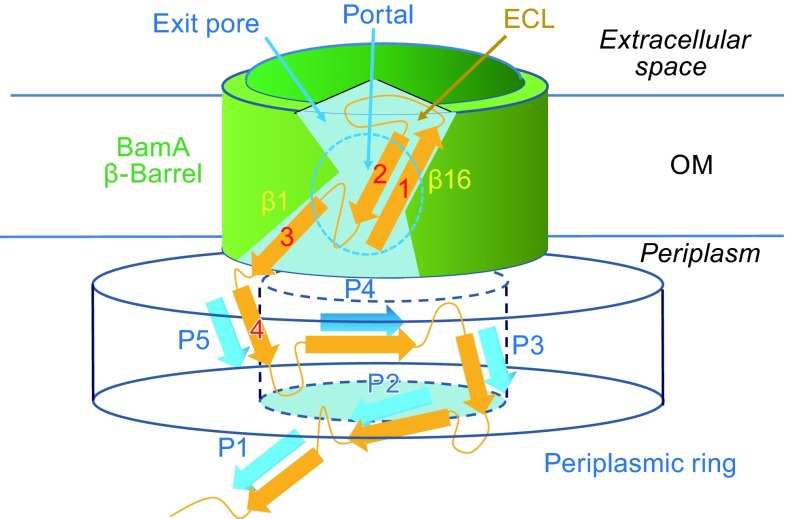
**Schematic of the BamA structure**. The BamA β-barrel is represented by a green cylinder. The edges of the β-sheets of the five POTRA domains (P1–P5) are represented by blue arrows. The nascent OMP is orange, and its β-strands are shown as arrows. The N-terminal strand is labeled “1”, and so on

Assembly of β-barrel integral MPs into the target membrane is catalyzed by insertases of the Omp85 superfamily. In *Escherichia coli*, the β-barrel assembly machinery (Bam) complex is an Omp85 superfamily member and contains five subunits, BamA–E (Gu et al., 2016; Han et al., 2016; Noinaj et al., 2015). While BamA itself is a 16-strand β-barrel protein (Noinaj et al., 2013), all other accessary proteins, BamB–E, are lipoproteins, each attaching to the inner leaflet of the OM via an N-terminal, triple-acylated moiety. BamA and BamD are essential parts of this complex, but other components are required for maximum OMP folding activity (Malinverni et al., 2006; Sklar et al., 2007a; Wu et al., 2005). The BamA β-barrel is partially closed from the extracellular side by a large capping dome consisting of extended inter-strand loops (mainly from the β11-β12 connection loop, ECL6), presumably to prevent membrane leakage. The cavity of the BamA β-barrel appears to be too small to house a fully folded OMP substrate, yet large enough to accommodate a couple of substrate β-hairpins (Noinaj et al., 2015). While most of the β-strands of BamA resemble those of a typical β-barrel, its C-terminal strand, β16, is short and kinks at its C-terminus. These structural features are evolutionarily conserved in the Omp85 superfamily. The β16 strand interacts only loosely with the β1 strand in the middle of the lipid bilayer. The corresponding region between the β1 and β16 strands is termed a portal or lateral opening (Fig. 1). In fact, this portal is the only transmembrane gap in the wall of the BamA β-barrel that allows the substrate peptide to exit from the cavity and enter the OM while avoiding topologic crossing-over between peptides of the substrate and the BamA β-barrel. Thus, the portal is proposed to be the exit through which the substrate β-strands are released. Meanwhile, an opening on the extracellular cap of the cavity (termed the exit pore) likely serves as the exit for ECLs of the substrate OMP (Noinaj et al., 2013).

BamA contains five periplasmic domains that are N-terminal to its β1 strand, and they are referred to as polypeptide transport-associated (POTRA) domains 1–5 (numbered sequentially from the N-terminus). Each of these POTRA domains contains a three-stranded β-sheet and two α-helices (with an order of β1-α1-α2-β2-β3) (Noinaj et al., 2013). Truncation experiments showed that only the last POTRA domain, POTRA-5, is essential for BamA function (Bos et al., 2007; Gessmann et al., 2014). POTRA1–4 are consistently missing in the mitochondrial Omp85 homolog SAM50 (Bohnert et al., 2015), while another Omp85 protein in *E. coli*, TamA, contains three POTRA domains (PDB ID: 4C00) (Gruss et al., 2013). In the recently published crystal structure of the BamA–E complex, POTRA1–5 form a right-handed spiral structure that is stabilized by the accessory proteins BamB–E (PDB ID: 5AYW) (Han et al., 2016). Together, POTRA2–5 and BamB–D form a ring near the periplasm-OM interface, with POTRA-1 located below the ring (Fig. 1). The periplasmic ring is ~40 Å thick and contains a chamber that is ~40 Å in diameter. Furthermore, near the periplasm-OM interface and the β1-β16 portal, there is an ~16 × 42 Å surface hole that connects the chamber of the periplasmic ring to the outside of the complex. This surface hole was proposed by the authors to be the exit for the OMP substrate (Han et al., 2016). Consistent with their essential roles in Bam functions, POTRA-5 and BamD have been observed to directly interact with each other and to participate in the formation of the surface hole. Interestingly, in the spiral structure of the five POTRA domains, all β-sheets are exposed to the solvent or the interior surface the chamber (Fig. 1). Furthermore, the β-sheet edges (i.e., the β2 strands) from the five POTRA domains form a nearly connected spiral track. This track runs from the bottom of the periplasmic ring all the way to the β1 strand of the BamA β-barrel. The distances between consecutive β-sheet edges (midpoint-to-midpoint) range from 22 Å to 36 Å in *E. coli* BamA. None of these edge-strands contains proline residues, which might potentially block β-sheet extension. The accessory proteins BamB–E probably function to stabilize this POTRA track. For instance, BamD stabilizes the connection between POTRA-5 and the β-barrel, as well as the POTRA1–2 connection. In addition, BamB stabilizes the POTRA2–3 connection, and BamE, together with BamD, stabilizes the POTRA4–5 connection. A similar, yet more flexible, arrangement of POTRA domains relative to the β-barrel has also been observed in TamA (PDB ID: 4C00), which has three POTRA domains (Gruss et al., 2013), and FhaC (PDB ID: 2QDZ), which has two POTRA domains (Clantin et al., 2007). In contrast to the complete complex, the spatial arrangement of the POTRA domains relative to the β-barrel is drastically different in the absence of the accessory subunits BamB–E (Noinaj et al., 2013). For instance, POTRA-5 blocks the periplasmic entry of the β-barrel in isolated BamA. Therefore, we believe that the newly published complete BamA–E complex is more informative in terms of understanding the β-barrel assembly mechanism. Intriguingly, in the crystal structure of *E. coli* BamA POTRA1–5 (PDB ID: 2QCZ), the β2 strand of POTRA-3 of one molecule interacts with a short peptide from POTRA-5 of a symmetry-related neighboring molecule through parallel β-strand hydrogen bonding (Kim et al., 2007). Similarly, in the crystal structure of TamA (PDB ID: 4BZA), POTRA-3 (corresponding to POTRA-5 of BamA) was found to interact in a parallel fashion with the β-sheet of POTRA-2 from another molecule via their β2 strands (Gruss et al., 2013). These structural observations suggest that the POTRA domains may bind the unfolded peptide substrate through β-sheet expansion (also called β-augmentation). It is probable that the POTRA track in the BamA–E complex provides a loading path for the unfolded peptide substrate (Bergal et al., 2016; Knowles et al., 2008), and the chamber of the periplasmic ring provides a path for ECLs to move from the periplasmic space into the cavity of the BamA β-barrel. Interestingly, the proposed binding modes between the nascent peptide and the β2 strands from the POTRA domains in the periplasmic ring are also in a parallel fashion (Fig. 1). This hypothesis can be tested by introducing proline residues at the β-sheet edges (i.e., the β2 stands) of POTRA domains. Each point mutation of proline-substitution would eliminate two potential hydrogen bonds for β-augmentation. A triple proline-mutation in the β2 strand of POTRA-5 of *E. coli* BamA consistently impaired cell growth (Gu et al., 2016). However, the mutation positions in this variant are in a registration that disrupts the three-strand β-sheet rather than directly blocks β-augmentation. In addition, for a BamA-like protein with multiple POTRA domains, a mutation in one or two β2 strands may not always be sufficient to have a noticeable effect on β-barrel assembly. In short, we propose that POTRA domains function essentially as a chaperone for the unfolded peptides of β-barrel OMPs.

To move across the aqueous environment of the periplasmic space that separates the inner and outer membranes, unfolded peptides of OMPs are escorted by chaperones to ensure that they remain assembly-competent (Thoma et al., 2015). Among the major chaperones in the periplasm, the 45-kDa SurA (survival protein A) has been shown to be directly involved in Bam-mediated OMP assembly (Behrens et al., 2001; Rollauer et al., 2015). A physical interaction between SurA and the N-terminal POTRA-1 of BamA has been reported (Bennion et al., 2010). In particular, Arg64 of POTRA-1, which faces the chamber entrance of the periplasmic ring of BamA, interacts with SurA. In fact, SurA is the only periplasmic chaperone that can be chemically cross-linked with BamA *in vivo* (Sklar et al., 2007b); in contrast, evidence of direct binding between SurA and other accessory Bam proteins (e.g., BamD) is lacking. SurA was found to bind specifically to peptides containing an Ar-X-Ar sequence motif (where Ar stands for an aromatic residue and X stands for any residue) (Goemans et al., 2014), which is common in β-barrel OMPs. The crystal structure of SurA contains four domains: a substantial N-terminal domain, two peptidylprolyl isomerase (PPIase) domains, and a C-terminal tail. Among them, the PPIase-1 domain was shown to bind the Ar-X-Ar motif of an unfolded peptide of OMP (Bitto and McKay, 2002; Xu et al., 2007). However, both PPIase domains have been shown to be dispensable for *in vivo* chaperone activity (Behrens et al., 2001). Intriguingly, a β-hairpin from the N-terminal domain forms a three-stranded β-sheet with the very C-terminal end of SurA, and formation of this β-sheet is essential for the chaperone activity of SurA (Chai et al., 2014). In particular, the length and β-strand propensity of the C-terminal peptide, but not its detailed amino acid sequence, are important for the chaperone activity. Furthermore, SurA has been shown to bind to β-hairpins, which are considered to be building blocks of β-barrels (Thoma et al., 2015). These observations suggest that the chaperone function of SurA requires β-augmentation with its substrate, rather than recognition of a specific sequence. Multiple SurA proteins may bind to potential β-strands from the unfolded peptide of a nascent OMP in a pearl-necklace fashion. SurA proteins are probably released sequentially from the substrate peptide when the latter is transferred to the downstream chaperone, the POTRA track. In this scenario, multiple spatially organized POTRAs may compete effectively with individual SurA for substrate binding. Hypothetically, the peptide of a β-barrel contains two types of β-hairpins: those consisting of odd-even strands and those consisting of even-odd strands. The difference between the two types lies in their hairpin loops. For an odd-even hairpin, the two β-strands connect to an ECL, which is usually longer than the periplasmic loop. Thus, it is possible that with a β-augmentation mechanism, SurA binds to only one type (e.g., odd-even) of hairpin in a specific orientation, based on the properties of the substrate, such as its connection loop and hydrophobic surface (e.g., the Ar-X-Ar motif), as well as the peptide direction.

Moreover, the assembly of a few OMPs, e.g., TolC and BamA, has been shown to be SurA-independent (Bennion et al., 2010). These OMPs usually contain large, auto-folded, periplasmic domains, which are located at their N-termini, as shown in BamA (Han et al., 2016), or attached to β-hairpins that are to be inserted into the OM, as exemplified by TolC (Koronakis et al., 2000). These soluble periplasmic domains may help to prevent aggregation of the unfolded peptide, in a manner that is similar to those of well-folded soluble proteins (e.g., maltose-binding protein), which help fused peptides to stay soluble during recombinant protein expression (Bell et al., 2013). The soluble periplasmic domains may also contain signals to deliver a β-barrel peptide(s) directly to the BAM complex without help from SurA.

Currently, two mechanisms of Bam-mediated OMP assembly have been proposed based on previously available structures of Bam components: the Bam-assisted and Bam-budding models (Rollauer et al., 2015). On one hand, the Bam-assisted model proposes that BamA creates a local, thinned lipid bilayer region near the portal, which helps the peptide of the OMP substrate to overcome the kinetic energy barrier during membrane insertion. Indeed, the β1-β16 junction has fewer hydrogen (H)-bonds than other inter-strand interactions in the BamA β-barrel. A molecular dynamic simulation suggests that BamA disrupts the lipid bilayer near the β1-β16 portal region (Noinaj et al., 2013). On the other hand, the Bam-budding model (Gruss et al., 2013) proposes that the BamA β-barrel uses its exposed edge β-strand, in particular the β1 strand, as a template for the substrate peptide to initiate barrel formation. The OMP peptide would continue to fold into the β-barrel until the barrel eventually buds off from BamA and is released into the OM. During this process, there is a transient BamA-substrate complex; thus, the Bam-budding model is also called a β-augmentation mechanism (Noinaj et al., 2015). Consistent with this mechanism, it has been demonstrated that a lateral opening of the β-barrel at the portal is essential for BamA function (Noinaj et al., 2014). Noticeably, the Bam-assisted and Bam-budding mechanisms are not mutually exclusive, and both are likely to play roles in OMP assembly.

**Figure 2:**
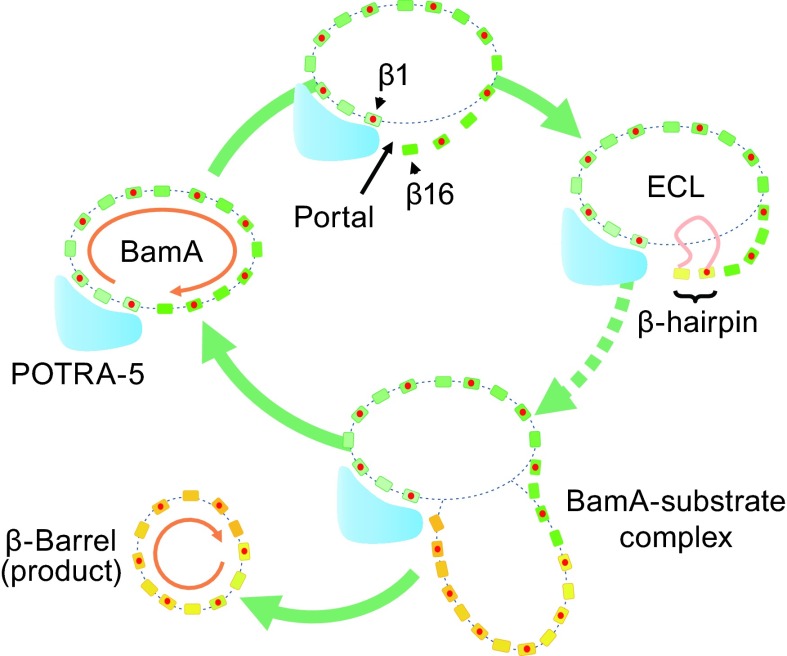
**Schematic of the mechanism of β-barrel assembly**. Each β-barrel is presented by a group of β-strands, and is viewed from the extracellular space. Each β-strand is represented by a rectangle, and those β-strands whose C-termini point to the extracellular space (i.e., odd-numbered strands) are marked with an additional red dot. The strands of BamA are colored light to bright green from the N- to C-termini, respectively. POTRA-5 (cyan) is N-terminal to the β1 strand, and it serves as the site for β-hairpin formation. The strands of the newly assembled substrate β-barrel are colored yellow to orange from the N- to C-termini, respectively. The orange curve represents an extracellular loop (ECL) of the β-barrel substrate. The overall rotation of the β-barrel peptide, as viewed from the extracellular space, is clockwise (brown arrows)

Interestingly, all known three-dimensional structures of β-barrel OMPs have a clockwise rotation when viewed from the extracellular space, the same pattern as that of the BamA β-barrel (Fig. 2). Based on the budding model, if the observed rotation direction of β-barrels is maintained as a general property of OMPs, then either the N-terminus of the OMP substrate initiates barrel building by binding to the β16 edge-strand of the BamA β-barrel, or the C-terminus of the OMP substrate first binds to the other edge-strand, β1. Accordingly, the gap in the transient super-barrel of the BamA-substrate complex must be associated with either the β1 or β16 strand of the BamA β-barrel. We hypothesize that while the BamA-substrate complex changes its shape as the super-barrel expands, the exposed edge of the growing barrel is likely to remain in a fixed position so that it is ready for further substrate loading. Such a feeding mechanism may be particularly important for the assembly of a β-barrel from multiple copies of the same subunit. For example, CsgG is an amyloid secretion channel that exports curli subunits for biofilm formation. Its crystal structure shows nine copies of the same four-stranded subunit, which forms a 36-stranded β-barrel, the largest one known to date (Cao et al., 2014). In this case, there is no continuous pulling force between the peptides of neighboring subunits. Therefore, the putative Bam-CsgG complex appears to have to start again when loading each new copy of the subunit, and it repeats the process nine times.

It is reasonable to hypothesize that the main function of the β1 strand, as well as POTRA-5, is to feed an unfolded peptide to the transient BamA-substrate super-barrel. This explanation may also apply to the essential role of the BamD subunit, which stabilizes the relative conformation between the BamA β-barrel and POTRA-5. In contrast, on the β16 side of the portal, there is no conserved, putative docking site for substrate peptides. In addition, because of a lack of interaction with the periplasmic ring (Han et al., 2016), the C-terminal β16 strand is more likely to be structurally flexible than the β1 strand. Therefore, we hypothesize that the opening of the growing BamA-substrate super-barrel remains located near the β1 strand, while strand β16 provides a template from which the neo-β-barrel initiates (Figs. 1 and 2). As pointed out above, this model would require the folding of an OMP β-barrel to start from its N-terminus by attaching to strand β16 of BamA. One of the benefits of such a model is that the folding process may be initiated before peptide translation and translocation are completed. This argument is supported by an early observation from a pulse-chase experiment in which the OM captured a large amount of incompletely translated nascent proteins (de Leij et al., 1979).

However, based on reports that the C-terminus of an OMP may contain a so-called β-signal that is important for assembly (Robert et al., 2006; Struyve et al., 1991), it is commonly assumed that the C-terminus of the substrate peptide initiates the binding with BamA. Nevertheless, there is no structural evidence for such a C-terminal initiation. In contrast, it was observed that either truncation of the first β-strand or mutations in the last β-strand in PhoE (a substrate of the Bam complex) reduced the assembly efficiency (Bosch et al., 1988; Struyve et al., 1991). In addition, disrupting the C-terminal β-signal did not prevent membrane insertion of the substrate, but led to the improper folding of substrates. In contrast to the common perception, our N-terminal initiation model predicts that building of the β-barrel of an OMP substrate starts at its N-terminus, while the C-terminal β-signal may be utilized for the proper termination of barrel expansion. A definite answer to the direction of β-barrel expansion in a BamA-substrate super-barrel remains to be established.

Right after transferring the substrate β-strands (or β-hairpins) from SurA to the POTRA track of BamA, the nascent peptide enters the chamber of the periplasmic ring and reaches the β1 edge-strand. Driven by both hydrophobic interactions with the lipid bilayer and H-bonding interactions with the neo-edge of the BamA-substrate super-barrel, the freshly bound peptide segments at the β1 strand and the POTRA-5 region form a β-hairpin, which further dissociates from the β1 strand and swings into the cavity of the BamA β-barrel, forming native H-hydrogen bonds with its N-terminal neighboring (i.e., even-numbered) β-strand. Note that the β16 strand serves as a template during the very beginning of the assembly process. The transient BamA-substrate super-barrel is expanded one hairpin at a time, with the hydrophobic face of the hairpin contacting the lipid bilayer and the hydrophilic ECL sliding through the partially capped, hydrophilic cavity of the super-barrel toward the extracellular side of the OM. Meanwhile, more of the unfolded peptide is pulled into the periplasmic chamber along the POTRA track. In summary, we propose that the Bam complex simply provides: (i) a spiral POTRA track that functions as a chaperone for binding the unfolded peptide of the OMP substrate; (ii) a template (i.e., the β16 strand) for initiation of β-barrel assembly; and (iii) a TM hydrophilic channel for the ECL of each β-hairpin building block. Such a mechanism would be similar to that of the insertion of α-helical TM proteins by the translocase YidC, in which TM helix-hairpins function as the units of membrane insertion (Cymer et al., 2014).
